# Reduction of *Baylisascaris procyonis* Eggs in Raccoon Latrines, Suburban Chicago, Illinois, USA

**DOI:** 10.3201/eid2012.140977

**Published:** 2014-12

**Authors:** Kristen Page, Timothy J. Smyser, Elise Dunkerton, Emily Gavard, Bruce Larkin, Stanley Gehrt

**Affiliations:** Wheaton College, Wheaton, Illinois, USA (K. Page, E. Dunkerton, E. Gavard, B. Larkin);; Purdue University, West Lafayette, Indiana, USA (T.J. Smyser);; State University of New York College of Environmental Science and Forestry, Syracuse, New York, USA (E. Gavard);; The Ohio State University, Columbus, Ohio, USA (S. Gehrt)

**Keywords:** Baylisascaris, landscape, management, prevention, zoonoses, raccoons, Illinois, parasites, roundworms, ascarids,

## Abstract

*Baylisascaris procyonis*, a common roundworm of raccoons, causes severe or fatal human infections, often in suburban areas. To evaluate the effectiveness of a baiting strategy requiring minimal labor, we distributed medicated baits near raccoon latrines in suburban Chicago, Illinois, USA. This strategy lowered *B. procyonis* prevalence in raccoons, possibly reducing risk to humans.

Modification of landscapes resulting in human-dominated ecosystems is one of the most important drivers of emerging diseases (*1*). In human-dominated landscapes, the transmission dynamics of diseases often change as host and pathogen population dynamics respond to loss or creation of habitat (*1*). As a result, contact rates can increase, and humans can be infected (*2*). Public health officials, veterinarians, and wildlife ecologists increasingly are focusing on mitigation strategies to decrease the potential for human disease in such landscapes (*2*).

*Baylisascaris procyonis* roundworms are ubiquitous ascarid parasites of raccoons; prevalence of infection can reach 82% (*3*). Infected raccoons can shed thousands of eggs daily; transmission generally occurs at raccoon latrines where fecal material accumulates (*4*). *B. procyonis* eggs are highly resistant and can remain viable in the environment for years (*3*). Thirty severe or fatal human cases of baylisascariasis have been reported (*5**–**7*; K.R. Kazacos, pers. comm), many of which occurred in human-dominated landscapes (K.R. Kazacos, pers. comm). Therefore, mitigation strategies appropriate for urban and suburban landscapes need to be developed.

Anthelmintic bait distribution has been evaluated for managing *B. procyonis* (*5*,*6*); however, to our knowledge, no *B. procyonis* mitigation study has been conducted in urban or suburban landscapes, where the risk for transmission to humans is highest. Additionally, previous studies implemented a regimen of latrine removal and substrate sterilization that might not be feasible for resource-restricted wildlife management officials. Furthermore, raccoon core habitat within suburban environments is fragmented with raccoon populations concentrated in preserves interspersed among residential areas (*8*). As a result, raccoons frequently move throughout adjacent residential areas, creating latrines in close proximity to homes and placing humans at risk to *B. procyonis* exposure (*9*). The ability of raccoons to exploit both natural and human-dominated landscapes can present challenges for *B. procyonis* mitigation, given that bait distribution is generally restricted to natural habitats (*10*). Our objective was to evaluate the effectiveness of a baiting strategy requiring minimal labor investment in a highly developed suburban region of Chicago, Illinois, USA. By modifying an existing mitigation strategy (*5*) and implementing it in a suburban landscape, we hope to provide wildlife managers and public health officials with a feasible strategy to decrease *B. procyonis* infection risks for children living in suburban landscapes.

## The Study

We conducted this study in Cook and McHenry Counties, Illinois, within the Chicago metropolitan area, a landscape characterized as 45% developed, 12% forested and open, 31% agricultural, 10% vacant or wetland, and 2% water (*11*) (Figure). Our experiment was conducted throughout 6 study sites associated with 6 forest preserves, 3 each in Cook and McHenry Counties. In September and October 2012, we sampled sixty-three 200-m^2^ quadrats across the 6 study sites. We sampled 8–16 quadrats per study site; the number of quadrats per site were related to the amount of forested habitat within each site (*12*). All fecal deposits were collected individually for analysis from each latrine within sampling quadrats (*13*). In contrast to previous studies (*5*,*6*), latrine substrates were not sterilized with a propane torch, nor were all latrines located and removed from the treatment sites. Similar to previous studies (*5*,*6*), we distributed anthelmintic baits monthly at 1.5 baits/hectare at each of the 3 randomly selected treatment sites for the duration (12 months) of the study. Baits were similar to oral rabies vaccine baits and consisted of a hollow fishmeal polymer food attractant (15 g, 33 mm × 32 mm × 21 mm) in which we placed 180 mg (base) of pyrantel pamoate mixed with marshmallow creme, which was sealed within the hollow chamber with paraffin wax. We distributed baits by hand along transects through forested portions of each site to achieve a relatively even distribution of 1.5/hectare. After 1 year of monthly bait distribution, we repeated latrine sampling in September and October 2013, using an equal sampling effort (sixty-three 200-m^2^ quadrats) during surveys before and after treatment. All fecal samples were stored at –20°C until they were examined for *B. procyonis* eggs. We used centrifugal fecal flotation in Sheather sugar solution and microscopic examination to evaluate all samples for eggs (*3*). Each fecal sample was determined to be positive or negative, and prevalence (proportion positive) was determined for each year, site, and treatment type. We evaluated differences in prevalence by year, site, and treatment type using χ^2^ analysis with a Fisher exact test. The Wheaton College Institutional Animal Care and Use committee approved this study.

**Figure:**
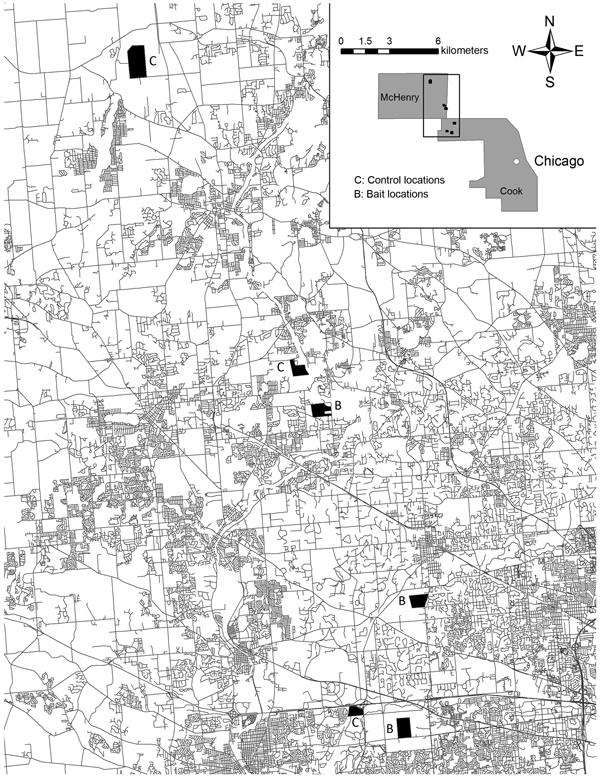
Location of study areas within the metropolitan area of Chicago, Illinois, USA, showing treatment and control sites. Insert indicates location of metropolitan Chicago counties where baits were placed.

We sampled 63 latrines (2.5 × 10^−5^/m^2^) in 2012, and 59 latrines (2.0 × 10^−5^/m^2^) in 2013. Latrine density did not differ between sampling years (*F* = 0.124, df = 1, p = 0.725). Pretreatment (2012) sampling of latrines resulted in 209 fecal samples, and a prevalence of 13% ± 4.56% across sites. The prevalence of *B. procyonis* roundworms did not differ between treatment (14% ± 6.91%) and control (12% ± 6.02%) sites before placement of anthelmintic baits (χ^2^ = 0.368, d.f. = 1, p = 0.544) (Table). Posttreatment sampling of latrines resulted in 124 fecal samples, and a prevalence of 11% ± 5.28% across sites. Prevalence across sites did not differ between years (χ^2^ = 0.44, d.f. = 1, p = 0.602) (Table); however, prevalence differed significantly between treatment (3% ± 3.94%) and control (21% ±1 1.07%) sites after treatment (χ^2^ = 11.28, d.f. = 1, p<0.001) (Table).

**Table:** Findings for baited and control locations in a study of prevalence of *Baylisascaris procyonis* eggs at raccoon latrines, suburban Chicago, Illinois, USA*

Location	2012 (before treatment)		2013 (after treatment)

Fecal samples, no. positive/total	Positive ± 95% CI, %		Fecal samples, no. positive/total	Positive ± 95% CI, %
All	27/209	13 ± 4.56		13/124	11 ± 5.28
Control areas	14/97	14 ± 6.91		11/52	21± 11.07
Baited areas	13/112	12 ± 6.02		2/72	3 ± 3.94

*Prevalence was determined as the proportion of positive fecal samples among all fecel sampled at a location. Baited locations received 1.5 baits/hectare.

## Conclusions

Previous strategies to decrease prevalence of *B. procyonis* roundworms required removing all latrines and heat sterilizing latrine substrates (*5*,*6*). Wildlife managers in urban or suburban settings often do not have the resources to implement such a labor-intensive strategy. We found that a modified strategy that eliminates latrine removal and sterilization but retains the monthly distribution of baits effectively reduced environmental contamination with *B. procyonis* eggs.

Implementation of this anthelmintic baiting strategy in suburban green spaces might significantly reduce risk for exposure by humans who use green spaces for recreation or live in close proximity to forested areas (*9*). However, both material and distribution costs can be substantial when such management action is implemented at landscapes scales. This study shows that the monthly distribution of baits and subsequent consumption by raccoons would keep reinfections from reaching patency (32–38 days) (*3*). For initial disease control, it is recommended that intervals between consecutive bait distributions not exceed the prepatency period (*14*). Once prevalence is reduced, interbaiting intervals can be extended while reduced prevalence is maintained (*14*). Although we implemented labor-intensive hand baiting, implementation of bait stations (*10*) could maintain management efficacy while reducing labor required for bait distribution. Bait stations have been used successfully for oral rabies vaccination in raccoons (*10*) and may enable more targeted bait distribution in areas of high raccoon density. Our study demonstrated that anthelmintic baiting successfully reduced environmental contamination with *B. procyonis* eggs; prevalence among treatment sites decreased nearly 80% with 1 year of treatment; however, further study is needed to identify optimal long-term bait distribution frequencies and bait distribution strategies to make anthelmintic baiting a viable and sustainable management solution for *B. procyonis* control.
